# Acute responses of circulating microRNAs to low-volume sprint interval cycling

**DOI:** 10.3389/fphys.2015.00311

**Published:** 2015-10-30

**Authors:** Shu Fang Cui, Wei Li, Jie Niu, Chen Yu Zhang, Xi Chen, Ji Zheng Ma

**Affiliations:** ^1^State Key Laboratory of Pharmaceutical Biotechnology, NJU Advanced Institute for Life Sciences (NAILS), School of Life Sciences, Nanjing UniversityNanjing, China; ^2^Jiangsu Engineering Research Center for MicroRNA Biology and Biotechnology, Nanjing UniversityNanjing, China; ^3^The Lab of Military Conditioning and Motor Function Assessment, The PLA University of Science and TechnologyNanjing, China

**Keywords:** plasma microRNAs, biomarkers, blood lactate, blood hormones, anaerobic capacity

## Abstract

Low-volume high-intensity interval training is an efficient and practical method of inducing physiological responses in various tissues to develop physical fitness and may also change the expression of circulating microRNAs (miRNAs). The purpose of the present study was to examine whether miRNAs for muscle, heart, somatic tissue and metabolism were affected by 30-s intervals of intensive sprint cycling. We also examined the relationship of these miRNAs to conventional biochemical and performance indices. Eighteen healthy young males performed sprint interval cycling. Circulating miRNAs in plasma were detected using TaqMan-based quantitative PCR and normalized to Let-7d/g/i. In addition, we determined the levels of insulin-like growth factor-I, testosterone and cortisol, and anaerobic capacity. Compared to plasma levels before exercise muscle-specific miR-1 (0.12 ± 0.02 vs. 0.09 ± 0.02), miR-133a (0.46 ± 0.10 vs. 0.31 ± 0.06), and miR-133b (0.19 ± 0.02 vs. 0.10 ± 0.01) decreased (all *P* < 0.05), while miR-206 and miR-499 remained unchanged. The levels of metabolism related miR-122 (0.62 ± 0.07 vs. 0.34 ± 0.03) and somatic tissues related miR-16 (1.74 ± 0.27 vs. 0.94 ± 0.12) also decreased (both *P* < 0.05). The post-exercise IGF-1 and cortisol concentrations were significantly increased, while testosterone concentrations did not. Plasma levels of miR-133b correlated to peak power (*r* = 0.712, *P* = 0.001) and miR-122 correlated to peak power ratio (*r* = 0.665, *P* = 0.003). In conclusion sprint exercise provokes genetic changes for RNA related to specific muscle or metabolism related miRNAs suggesting that miR-133b and miR-122 may be potential useful biomarkers for actual physiological strain or anaerobic capacity. Together, our findings on the circulating miRNAs may provide new insight into the physiological responses that are being performed during exercise and delineate mechanisms by which exercise confers distinct phenotypes and improves performance.

## Introduction

Sprint interval training, interspersed with sufficient recovery periods (2–4 min), can produce the best possible average sprint performance over a series of sprints (<45 s; Weston et al., 2014). Therefore, sprint interval training is a commonly used intervention to maintain skeletal muscle health and to improve exercise performance, especially in individuals who require a high contribution of glycolytic energy (e.g., track-and-field sprint athletes and some team sport athletes; Buchheit and Laursen, 2013). Similarly, sprint interval cycling (SIC), which is modeled on the Wingate Anaerobic test, is extremely demanding and involves all-out 30-s sprints interspersed with 4 min active recovery periods with no resistance (Burgomaster et al., 2005). The high-intensity nature of SIC is thought to recruit all types of muscle fibers (Weston et al., 2014). However, repeated all-out 30-s bouts of exercise separated by 4 min of rest increasingly depend on aerobic metabolism (Burgomaster et al., 2005). Exercise intensity places mechanical and/or metabolic stress on contracting muscles (Laursen, 2010). The anabolic (e.g., growth hormone, testosterone, and insulin-like growth factor-1) and catabolic (e.g., cortisol) processes of tissue remodeling following exercise loading are typically reflected by acute changes in hormonal concentrations (Schoenfeld, 2012).

The microRNAs (miRNAs) are typically small, ~19–22 nucleotides long, non-coding RNA molecules that post-transcriptionally regulate gene expression by base-pairing with the 3′ untranslated region of complementary messenger RNA targets (Bartel, 2004). Recently, some miRNA species (particularly muscle-specific miRNAs and inflammatory-related miRNAs) have been found to change in human serum/plasma after acute prolonged endurance training, eccentric exercise, and strength exercise (Baggish et al., 2011, 2014; Aoi et al., 2013; Banzet et al., 2013; Gomes et al., 2014; Mooren et al., 2014; Uhlemann et al., 2014). Studies have suggested that they can be used as potential biomarkers for aerobic capacity and as markers or mediators of physiological adaptations (Baggish et al., 2011, 2014; Aoi et al., 2013; Banzet et al., 2013; Mooren et al., 2014; de Gonzalo-Calvo et al., 2015).

Exhaustive exercise has a deep effect on cellular, humoral, and metabolic processes of the body (Spencer et al., 2005; Meckel et al., 2011; Gibala et al., 2012). We hypothesized that a single session of low-volume sprint interval cycling can change the circulating miRNAs (c-miRNAs) profile. Accordingly, the levels of the myomiR family (miR-1, miR-133a, miR-133b, and miR-206) and other muscle-specific miRNA (miR-499), metabolism-related miRNA (miR-122), and somatic tissues-related miRNA (miR-16) were evaluated in response to extreme SIC. In addition, to provide insight into the potential roles of these c-miRNAs, we also determined whether alterations in the levels of these miRNAs in response to extreme SIC are correlated with changes in conventional anabolic-catabolic hormonal biomarkers or anaerobic capacity.

## Materials and methods

### Ethical approval and participants

Eighteen healthy young male participants (age, 20.23 ± 0.97 years; height, 1.75 ± 0.06 m; body mass, 68.90 ± 8.83 kg; and BMI, 22.39 ± 2.06 kg·m^−2^) who were habituated to a regular exercise regimen were recruited to participate in this study. University cadets were contacted to volunteer for this study who led similar lives and who had the same dietary habits for 2 years prior to the study. None of the subjects had any current or prior chronic disease, a history of smoking or current use of any medications. Written informed consent was obtained from all of the participants. Ethical approval for this study conformed to the standards of the Declaration of Helsinki, and the protocol was approved by the Institutional Review Board of Nanjing University.

### Study design

Before the SIC session, the participants refrained from exercise for at least 72 h. Sprint interval cycling was performed on a mechanically braked stationary cycle ergometer (Monark Ergomedic 839E, Monark, Sweden) against a pre-determined force load of approximately 7.5% of the subject's body weight in kilograms. Sprint interval cycling involved two 30-s all-out sprints (Sprint 1 and Sprint 2) with 4 min of active recovery between them (Burgomaster et al., 2005). The tests were conducted between 10:00 am and 11:30 am.

### Anaerobic capacity

The first 30 s of Wingate data for each participant were used to assess anaerobic power. Fatigue index was calculated as ([Peak Power − Minimum Power]/Peak Power) × 100%. The change in Wingate data for Sprint 1 and Sprint 2 were used to assess the ability to maintain anaerobic power.

### Plasma sampling

Venous blood was collected at two different time points during the acute exercise test. Five milliliters of blood was collected in standard anticoagulant (EDTA)-treated Vacutainer tubes prior to acute exercise testing (Pre) and within 1 min of exercise testing completion (Post). All of the blood samples were centrifuged at 1500 × g for 10 min to pellet cellular elements immediately after each blood draw and were then centrifuged at 10,000 × g for 5 min at 4°C to completely remove cell debris. To minimize freeze–thaw degradation, the supernatant plasma was then aliquoted and immediately frozen at −80°C.

### Blood lactate and hormones

The blood lactate (LA) concentration was measured with an automatic lactate analyzer (EKF Diagnostic, Germany). Blood samples were analyzed for conventional physiological markers, including lactate, insulin-like growth factor-1 (IGF-1), testosterone, and cortisol. The IGF-1 level was measured with an IMMULITE 2000 Analyzer (EuroDPC Med Limited, Llanberis, UK). The assays were conducted using the solid-phase, enzyme-labeled, chemiluminescent immunometric method in accordance with the manufacturer's instructions. The testosterone and cortisol levels in the plasma were determined by a chemiluminescence immunoassay (UniCel DxI 800, Access Immunoassay System, Beckmann Coulter GmbH, Krefeld, Germany). The IGF-1, testosterone and cortisol levels were considered normal according to the reference ranges for ages provided by the kit manufacturer.

### Mirna isolation and RT-qPCR

Total RNA, including miRNAs, was isolated from the plasma samples using a 1-step phenol/chloroform purification protocol as previously described (Liu et al., 2011). A panel of miRNAs was investigated that are related to either skeletal/heart muscle (miR-1, miR-133a, miR-133b, miR-206, and miR-499) or to metabolism and somatic tissue (miR-122 and miR-16; Aoi et al., 2013; Boettger et al., 2014; Bandiera et al., 2015). To quantify the abundance of mature miRNAs, real time quantitative polymerase chain reaction (RT-qPCR) was performed. RT-qPCR was performed using a TaqMan PCR Kit and an Applied Biosystems 7300 Sequence Detection System as previously described (Wu et al., 2014). The cycle threshold (Ct) data were determined using default threshold settings, and the mean Ct was determined from triplicate PCRs. In addition, we calculated the Ct values of Let-7d, Let-7g, and Let-7i because the use of this combination of reference genes (Chen et al., 2013) in human serum for normalization has been demonstrated to be superior to that of the other commonly used single reference genes. The relative levels of miRNAs were normalized to a Let-7d/g/i trio and were calculated using the 2^−Δ*ΔCt*^ method. ΔCt was calculated by subtracting the Ct values of Let-7d/g/i from the average Ct values of the target miRNAs. ▵Ct values were then compared (ΔΔCt) with each participant's own resting baseline at the Pre time point (normalized to fold change of 1).

### Statistical analyses

GraphPad Prism 5 and SigmaPlot 10.0 packages were used. Subject characteristics, exercise testing data and blood parameters were reported as the mean ± standard deviation, and c-miRNA data were presented as the mean ± standard error of the mean (SEM). Paired variables were compared in Student's *t*-test or a Wilcoxon's matched pairs test as appropriate for the data distribution. Correlation analyses were performed using the Spearman or Pearson's method as appropriate for the data distribution. Values of *P* < 0.05 were considered significant.

## Results

### Wingate performance

After the SIC, Wingate peak power was significantly decreased by 11% following two 30-s periods of all-out SIC (659.48 ± 217.64 W vs. 562.78 ± 146.38 W, *P* = 0.006). The average power was also significantly decreased by 16%. The average power of Sprint 1 was 6.69 ± 0.78 W·kg^−1^ and that of Sprint 2 was 5.58 ± 0.80 W·kg^−1^ (*P* < 0.001). There was no significant difference in the fatigue index between the first and second 30-s sprint (62.87 ± 11.22% vs. 60.26 ± 12.44%, *P* = 0.820). In addition, there was no significant difference in maximum speed between the first and second sprint (151.96 ± 16.83 rpm vs. 150.57 ± 20.04 rpm, *P* = 0.740).

### Blood lactate

After exercise, the LA concentration significantly increased (1.56 ± 1.90 mmol·L^−1^ vs. 11.27 ± 1.90 mmol·L^−1^, *P* < 0.001).

### Plasma hormones

Compared to plasma levels before exercise, the post-exercise IGF-1 concentration was significantly increased by 13% following two 30-s periods of all-out SIC (298.94 ± 50.13 ng·mL^−1^ vs. 332.72 ± 57.76 ng·mL^−1^, *P* < 0.001). Testosterone concentrations were similar between pre- and post-exercise (40.59 ± 8.45 nmol·L^−1^ vs. 41.74 ± 11.57 nmol·L^−1^, *P* = 0.608). The cortisol concentration increased by 45% (216 ± 87 nmol·L^−1^ vs. 278 ± 91 nmol·L^−1^, *P* = 0.028) and a decrease in testosterone/cortisol ratio tended to be statistical significant.

### The plasma miRNA levels in response to an acute bout of sprint interval cycling

**Figure 1:**
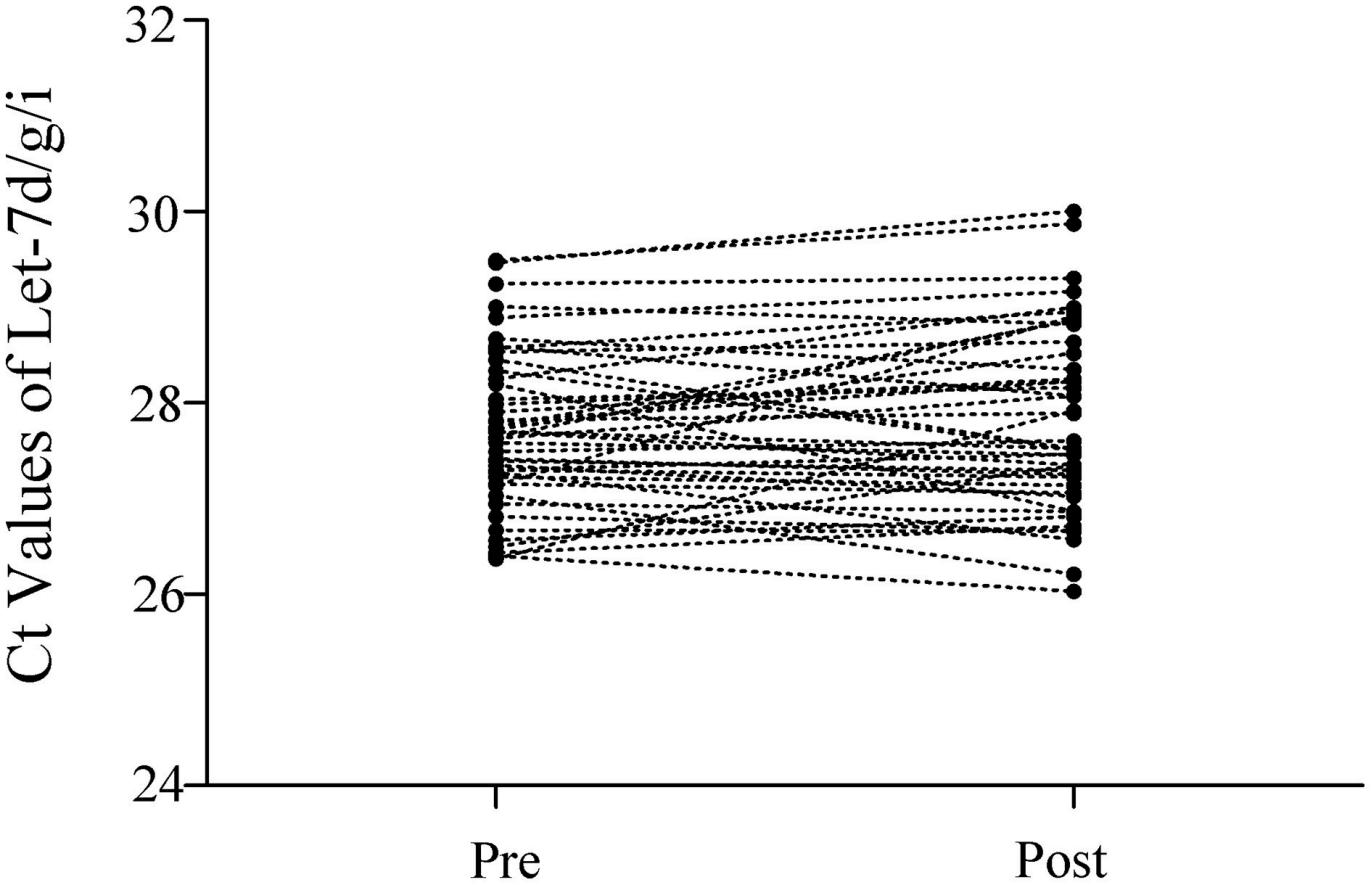
**The Ct values of Let-7d/g/i in plasma samples before and after the sprint interval cycling**. The total amount of Let-7d/g/i trio was simultaneously measured in a same RT-qPCR reaction. Let-7d, Let-7g, and Let-7i were reverse-transcribed in a single reaction using a mixture of stem-loop primers of Let-7d, Let-7g, and Let-7i (in the ratio of 1:1:1). Accordingly, real-time PCR was performed using a TaqMan miRNA probe pool of Let-7d, Let-7g and Let-7i (in the ratio of 1:1:1). Ct values of every individual's Let-7d/g/i before (Pre) and after (Post) the exercise almost remained unchanged.

**Figure 2:**
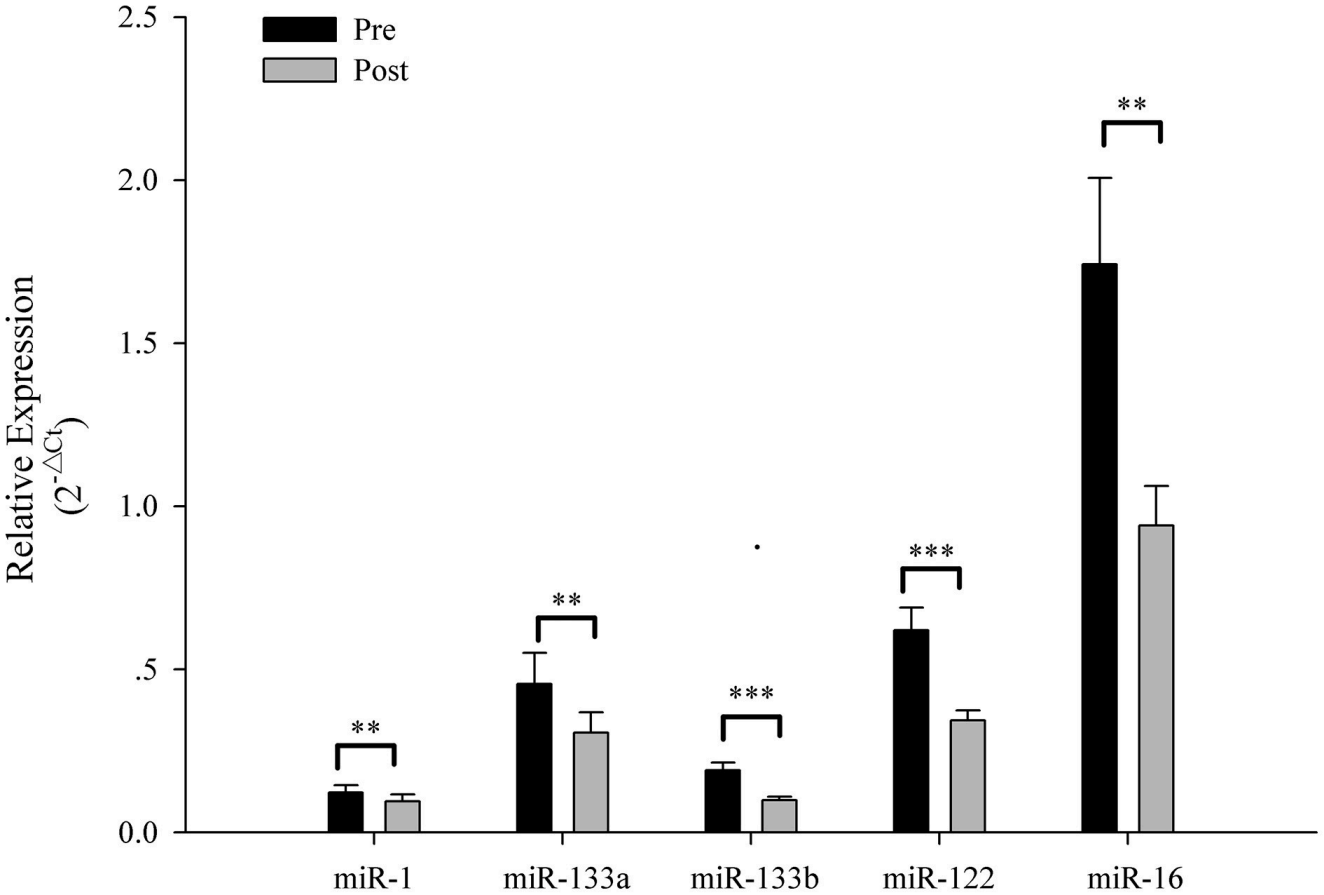
**The plasma miRNA levels in response to sprint interval cycling**. Plasma levels of miR-1, miR-133a, miR-133b, miR-122, and miR-16 decreased significantly after an acute bout of sprint interval cycling. Values represent the mean ± SEM obtained from 18 subjects. ^**^P < 0.01, ^***^P < 0.001.

**Figure 3:**
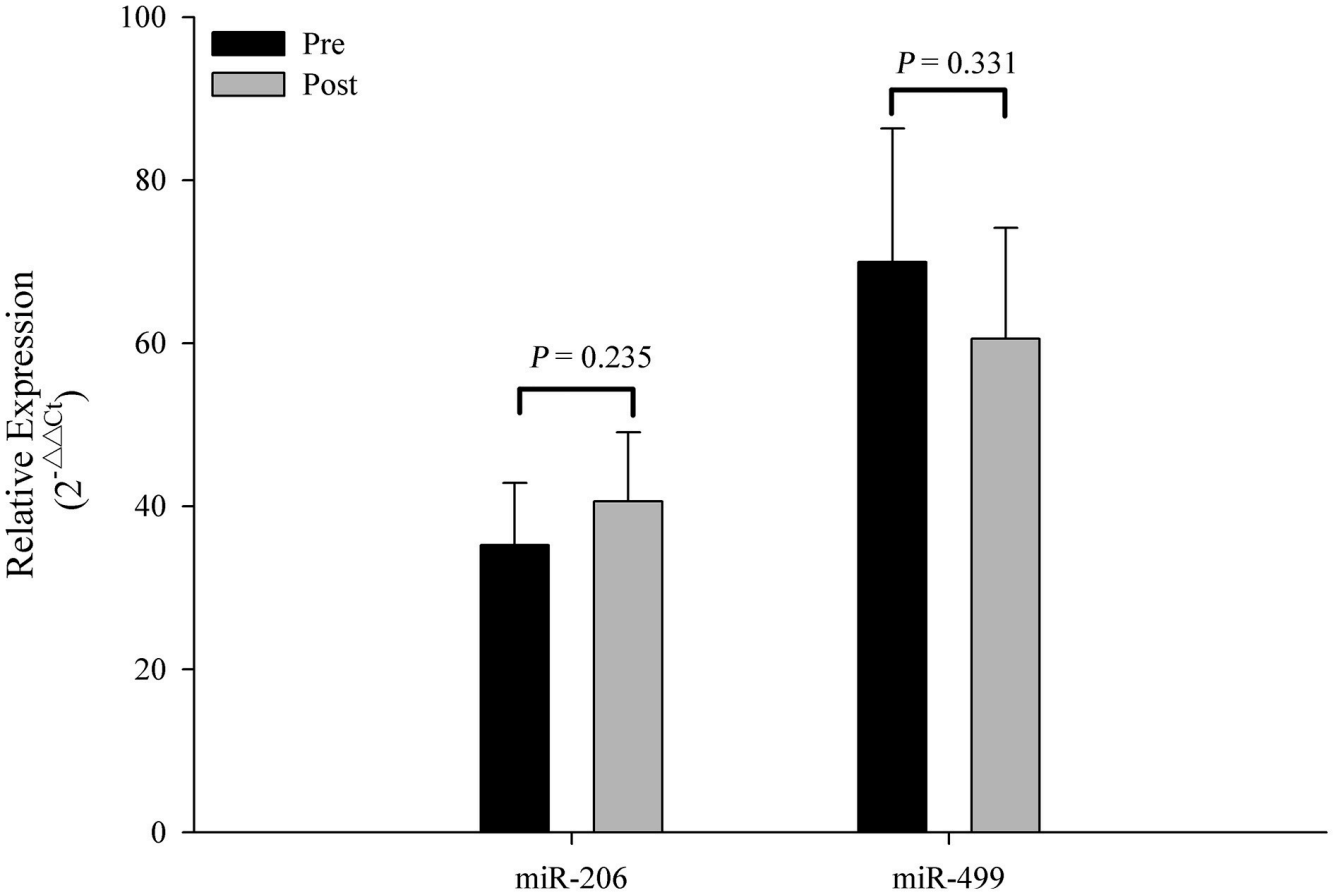
**Changes in circulating miR-206 and miR-499 in response to acute exercise**. Plasma levels of miR-206 and miR-499 showed no significant difference after an acute bout of sprint interval cycling. Values represent the mean ± SEM obtained from 18 subjects.

The Ct values of Let-7d/g/i at pre- and post-SIC show low variability (Figure 1; *P* = 0.280). Acute sprint interval cycling significantly decreased the levels of miR-1, miR-133a, miR-133b, miR-122, and miR-16 (Figure 2). Levels of miR-206 and miR-499 were not significantly changed by acute sprint interval cycling (Figure 3).

### Correlations between blood, anaerobic parameters and c-miRNA levels

To explore the feasibility of using c-miRNAs as biomarkers of an acute exercise response, the association of specific changes in c-miRNA was examined in relation to the changes in blood hormonal and anaerobic parameters. No correlations were found between the changes in the plasma IGF-1, testosterone, and cortisol levels, or the testosterone/cortisol ratio and the changes in the miR-1, miR-133a, miR-133b, miR-122, and miR-16 levels (Table 1).

**Table 1:** **Correlations between changes in exercise-related blood parameters and anaerobic parameters and changes in plasma levels of miRNAs (*n* = 18)**.

	**IGF-1**		**Testosterone**		**Cortisol**		**Testosterone/Cortisol**		**Peak Power of Sprint 1**		**Average Power of Sprint 1**		**Peak Power Ratio of Sprint1/Sprint2**		**Fatigue Index**	
	***r***	***P***	***r***	***P***	***r***	***P***	***r***	***P***	***r***	***P***	***r***	***P***	***r***	***P***	***r***	***P***
miR-1	−0.101	ns	−0.060	ns	−0.111	ns	0.165	ns	0.410	ns	−0.018	ns	−0.057	ns	0.201	ns
miR-133a	−0.379	ns	0.078	ns	−0.155	ns	0.060	ns	0.441	ns	−0.145	ns	−0.078	ns	−0.087	ns
miR-133b	−0.179	ns	0.086	ns	0.284	ns	−0.118	ns	0.712	0.001	0.076	ns	−0.053	ns	−0.059	ns
miR-122	−0.045	ns	0.023	ns	0.037	ns	0.009	ns	0.257	ns	−0.132	ns	0.655	0.003	0.012	ns
miR-16	0.066	ns	−0.137	ns	−0.143	ns	0.102	ns	0.189	ns	0.050	ns	−0.104	ns	0.096	ns

ns, not significant.

**Figure 4:**
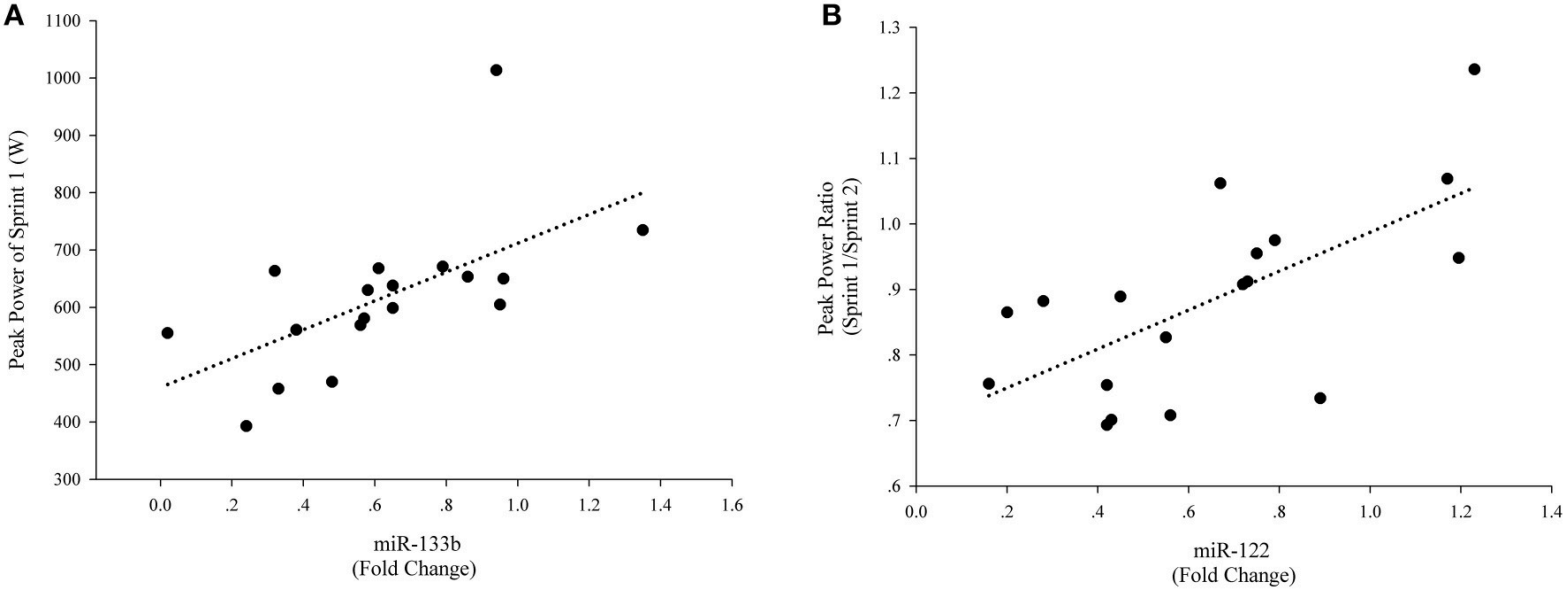
**Correlations of plasma miRNAs and exercise-related anaerobic parameters**. For each participant, baseline c-miRNAs levels before the exercise were assigned a fold change of 1, to which measurements obtained after the exercise were compared. A direct correlation was observed between levels of peak power of Sprint 1 and levels of plasma miR-133b (Post exercise) **(A)** A direct correlation was also observed between the peak power ratio of Sprint 1/Sprint 2 and levels of miR-122 (Post exercise) **(B)**.

There were not correlations among the levels of peak power, average power and fatigue index and levels of plasma miR-133a, miR-1, and miR-16 levels (Table 1). However, there was a significant correlation between the levels of peak power of Sprint 1 and levels of plasma miR-133b (Figure 4A). These results suggest a potential role for plasma miR-133b as a marker of anaerobic capacity. Furthermore, there was a significant correlation between the levels of the peak power ratio of Sprint 1/Sprint 2 and plasma miR-122 level (Figure 4B), suggesting a potential role of plasma miR-122 as a restriction marker of anaerobic capacity.

## Discussion

Sprint interval cycling is one of the most frequently used training methods in anaerobic sports. Our data indicated that several miRNA species in plasma responded to sprint interval cycling. These acute responses may be more critical to tissue growth and remodeling than chronic changes in resting concentrations (Kraemer and Ratamess, 2005). Exercise rapidly and transiently regulates several miRNA species in circulation, suggesting that they could be used to precisely monitor physiological acute responses to exercise.

Sprint interval exercise involves very high intensity, and the myosin heavy chain (MHC) IIa and IIx fibers are highly responsive to intense exercise at the transcriptional level for genes involved with muscle growth and remodeling (Trappe et al., 2015). miR-1 and miR-133a are expressed in both skeletal and cardiac muscles (Boettger et al., 2014). miR-133b and miR-206 are specific to skeletal muscle and preferentially detected in slow myofibers (Boettger et al., 2014). Pervious study has shown that circulating plasma miR-1 levels were significantly decreased in patients with supraventricular tachycardia, while miR-133 significantly increased in patients with ventricular tachycardia (Sun et al., 2015). The interactions of miRNAs (such as miR-1 and miR-133) with ion channel-encoding genes and calmodulin regulate cardiac contractility, rhythm and excitement (Terentyev et al., 2009). However, cardiac contraction during exercise is the physiological process most sensitive to calmodulin integrity, which can be affected by acute exercise (Sondergaard et al., 2015). Moreover, extracellular miRNAs are dynamic indices of pathophysiological processes in skeletal muscle (Roberts et al., 2013). Endurance also increased the miR-1 and miR-133a levels both in plasma and skeletal muscle (Nielsen et al., 2010; Baggish et al., 2014; Gomes et al., 2014; Mooren et al., 2014). During high intensity exercise the heart rate can greatly increase. In the present study, miR-1 and miR-133a levels in plasma decreased, and miR-1 and miR-133a expression in adult mice decreased during skeletal muscle hypertrophy (Mccarthy and Esser, 2007). These results indicate that these miRNAs may partly reflect the cardiac or skeletal muscle responses induced by exercise or a temporal regulation controlled by miRNAs during exercise.

Furthermore, miR-133b, miR-206, and miR-499 levels in plasma decreased or remained unchanged in the current study but significantly increased after acute endurance exercise in a previous study (Mooren et al., 2014). Training with high intensity exercise induces expression of fast-twitch fibers. Therefore, no change of circulating miR-206 and miR-499 observed in the present work, associated with slow myofibers or oxidative red fibers (Gan et al., 2013; Boettger et al., 2014), might reflect a non-predominance of slow-twitch fibers in SIC. However, at present, specific miRNAs related to fast-twitch fibers in human, such as MHC IIa and MHC IIx muscle fibers, have not been found.

Two miRNAs not restricted to muscle in origin (miR-122 and miR-16) also deceased in plasma following acute high intensity exercise. A previous study has shown that miR-122 is a key factor in liver development, differentiation, homeostasis, and metabolic function (Bandiera et al., 2015). The peroxisome proliferator-activated receptor (PPAR) γ coactivator 1-α (PGC-1α) plays a pivotal role in the regulation of the expression of mitochondrial proteins cytochrome c and cytochrome oxidase subunit I in the liver in response to a single exercise bout (Haase et al., 2011). Exercise induced beneficial alterations in the liver mitochondrial morphology and increased mitochondrial biogenesis (PGC-1α and mitochondrial transcription factor A) (Santos-Alves et al., 2015), and liver AMP-activated protein kinase (AMPK) activity increased during heavy exercise (Carlson and Winder, 1999). The putative effectors of miR-122-mediated metabolic control in the liver may be involved in both circadian metabolic regulators of the PPAR family and AMPK (Bandiera et al., 2015). Thus, the currently observed decrease of circulating miR-122 might reflect a liver cellular temporal regulation controlled by miRNAs during exercise. In addition, circulating miR-16 level also decreased, suggesting that non-muscle tissue is also needed to cope with this stress.

Exercise is a potent stimulus for the release of many hormones in response to the specific demands of the particular exercise type (Stokes et al., 2013). Changes in the anabolic-catabolic hormonal balance were found following brief sprint interval exercise training, may be used to gauge training adaptation to different anaerobic exercises (Kraemer and Ratamess, 2005; Meckel et al., 2011). But in the present study, there was no significant correlation between the change in these plasma hormone levels and the change in the c-miRNAs levels. Given their different physiological roles, it is likely that the c-miRNAs would show different expression patterns induced by SIC. However, in our study a negative correlation was observed between the reduced magnitudes in miR-133b and peak power and between the reduced magnitudes in miR-122 and the ability to maintain anaerobic power. In this case, the reduction in miR-133b and miR-122 in circulation may be considered a biomarker that reflects internal physiological stress caused by SIC.

At present, the mechanism(s) and clinical significance of an exercise-induced decrease in specific c-miRNAs remains poorly understood. An exercise-induced miRNA uptake by certain recipient cells has been postulated (Aoi et al., 2013; Aoi and Sakuma, 2014). Given the transportability of vesicles, the role of miRNAs in exosomes is gaining increasing attention. In the present study, high-intensity exercise may destroy exosomes, leading to degradation of miRNAs by RNases. However, the miR-206 and miR-499 in circulation remained unchanged, suggesting that significant degradation of exosomes did not occur as a result of SIC (Aoi et al., 2013; Aoi and Sakuma, 2014). The miRNAs in the exosomes are selectively packaged rather than included indiscriminately (Etheridge et al., 2013; Zhang et al., 2015). Moreover, the exosomal miRNA expression levels are altered under different physiological conditions (Etheridge et al., 2013; Zhang et al., 2015). It is reasonable to consider that acute high-intensity exercise may promote the uptake of some c-miRNAs into certain recipient cells (Aoi et al., 2013; Aoi and Sakuma, 2014). Further investigation is required to validate this hypothesis.

In conclusion, our data demonstrate that extreme sprint interval cycling affects the expression patterns of plasma miRNAs. Selective c-miRNAs, such as miR-133b and miR-122, may be potentially suitable for use as novel biomarkers to monitor training-induced acute changes within diverse tissues in response to low-volume sprint interval cycling.

## Author contributions

SC designed the study, performed the experiments, collected and analyzed the data, and revised the final version of the manuscript. WL and JN recruited participants, collected samples and performed the experiments. CZ and XC designed the study and critically revised the final version of the manuscript. JM designed the study, recruited participants, analyzed the data and wrote the final version of the manuscript. All authors read and approved the final version of the manuscript.

## Funding

The authors acknowledge that this work was supported by the National Basic Research Program of China (2014CB542300), the National Natural Science Foundation of China (81101330, 31271378, 81250044), the Natural Science Foundation of Jiangsu Province (BK2012014) and the Research Special Fund for Public Welfare Industry of Health (201302018). This work was also supported by the Program for New Century Excellent Talents in University from Ministry of Education of China (NCET-12-0261).

### Conflict of interest statement

The authors declare that the research was conducted in the absence of any commercial or financial relationships that could be construed as a potential conflict of interest.
